# Monomeric Glycine oxidase from *Azotobacter vinelandii for* Glycine biosensing

**DOI:** 10.1007/s11274-025-04657-4

**Published:** 2025-11-11

**Authors:** Aaron Mena-Rodríguez, Raul Garcia-Morales, Oscar González-Davis, Rafael Vazquez-Duhalt, Alejandro Huerta-Saquero, Andrés Zárate-Romero

**Affiliations:** 1 Centro de Nanociencias y Nanotecnología, Universidad Nacional Autónoma de México, Ensenada, B. C 22800 México; 2 Secretaría de Ciencia, Tecnología e Innovación, Alc. Benito Juárez, Humanidades, CDMX 03940 México; 3 División Académica de Ciencias Básicas, Universidad Juárez Autónoma de Tabasco, Cunduacán, Tabasco 86690 Mexico

**Keywords:** Monomeric glycine oxidase, Azotobacter vinelandii, Glycine biosensor, Enzymatic characterization

## Abstract

Glycine level is an indicator of diseases related to the central nervous system and metabolic disorders such as diabetes and obesity. It is, therefore, relevant to have tools that can detect glycine at physiological concentrations. Biosensors are a reliable alternative to chemical methods for glycine detection, with low cost and high response speed. In the case of glycine detection, a glycine oxidase (GO) can be used as the molecular recognition element in a biosensor. The GO from *Azotobacter vinelandii* is an uncharacterized enzyme with high sequence identity to the only monomeric FAD-dependent GO from *P. putida* KT2440. The monomeric state is favorable for oriented immobilization of active sites in a biosensor, which is attractive for future applications. This work characterized the GO from *A. vinelandii*, and a second-generation glycine amperometric biosensor was assembled. The enzyme was recombinantly expressed and purified. The oligomeric state of the enzyme was also confirmed as the monomer. Therefore, a substrate inhibition behavior was detected in the enzyme kinetics experiments with parameters *K*_m_=4.65, *k*_cat_=0.38, and *k*_si_=23.0. Two second-generation biosensors were assembled, and electrochemical characterization for the detection of glycine was carried out by chronoamperometry. The linear ranges of the biosensors (0.1 to 3 mM and 0.1 to 5 mM, for one- or two-enzyme layers respectively) are in the range of concentration of physiological fluids such as urine and sweat. This research opens new opportunities for applying new strategies that involve monomeric FAD-dependent glycine oxidases in glycine biosensing.

**Supplementary Information:**

The online version contains supplementary material available at 10.1007/s11274-025-04657-4.

## Introduction

The α-amino acid glycine is a major constituent in extracellular structural proteins in animals. It is the smallest amino acid in nature and does not possess chirality (Wang et al. 2013). Glycine is a biosynthetic precursor of several metabolites (glutathione, creatine, purines, heme, and serine) and is also a neurotransmitter in the central nervous system, acting as a co-agonist, together with D-serine, of glutamate and glutamatergic N-methyl-D-aspartate receptors (NMDAR) in the forebrain. Dysregulation of glycine levels affects health in certain diseases, such as non-ketotic hyperglycinemia (NKH), a severe autosomal recessive neurometabolic disorder characterized by glycine accumulation in fluids and body tissues, including the brain. In the severe form of NKH, the symptoms include spasticity, epileptic encephalopathy, absent psychomotor development, and therapy-resistant epilepsy (Shelkowitz et al. 2022).

Detecting glycine in blood and physiological fluids can provide information for diagnosing specific diseases and monitoring patient treatment (Sandlers 2020). The healthy basal glycine concentrations in plasma/blood range from 147 to 299 µM for males and 100–384 µM for females, and increase to 450–2363 µM in unhealthy levels (Pérez-Ràfols et al. 2020). In clinical settings, glycine is typically measured using liquid chromatography, a method known for its precision and reproducibility. However, it relies on costly instrumentation and demands skilled operators. Alternatively, commercial fluorometric assay kits are available, offering high sensitivity and specificity. Despite these features, they come with limitations, such as the need for sample dilution, multiple preparation steps, reliance on sequential enzymatic reactions, and the requirement for a fluorometer (Pérez-Ràfols et al. 2020). As a promising alternative, electrochemical biosensors offer several advantages, such as rapid detection, low-cost manufacturing, and the potential for miniaturization. There are some successful examples of these features in biosensors for glucose, uric acid, and L-lactate (Kim et al. 2019).

**Scheme 1:**
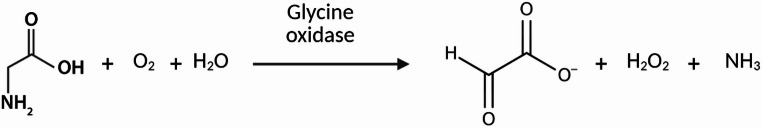
Reaction catalyzed by the glycine oxidase

Glycine oxidases (GOs) are enzymes that catalyze the conversion of glycine to glyoxylate, H_2_O_2,_ and NH_3_ in the presence of molecular oxygen (Scheme 1). The enzyme produces glycine imine, which is later hydrolyzed to glyoxylate and H_2_O_2_. In the genus *Bacillus*, the production of glycine imine is the first step for the synthesis of the thiazole moiety of thiamine (Settembre et al. 2003). Most of the characterized enzymes are tetrameric FAD-dependent enzymes. There are several studies discussing the enzymatic and structural properties of some glycine oxidases, such as *Bsu*Go from *Bacillus subtilis* (Settembre et al. 2003), *Bli*GO from *Bacillus licheniformis* (Zhang et al. 2016), *Bce*GO from *Bacillus cereus* (Zhan et al. 2013), and GOXK from *Geobacillus kaustophilus* HTA 426 (Martínez-Martínez et al. 2008). Additionally, the reported glycine oxidases can catalyze the oxidative deamination of secondary amines such as sarcosine and also D-amino acids. Sarcosine is a small endogenous amino acid that is present in blood and urine in low concentrations (Rosini et al. 2014). It has been proposed that the measurement of its concentration can be used as a biomarker in the progression of prostate cancer, as sarcosine levels are increased in metastatic prostate cancer cells (Sreekumar et al. 2009; Khan et al. 2013).

The use of GO for glycine biosensing has been reported recently. A fluorescence biosensor was developed using an engineered GO from *B. subtilis*, which presents an 11-fold *k*_cat_/*K*_m_ value compared to the wild-type (Rosini et al. 2014). Conversely, a different type of glycine oxidase dependent on a cysteine tryptophylquinone cofactor has also been characterized. This GO from *Marinomonas mediterranea* showed a higher *k*_cat_ for glycine than FAD-dependent GOs. An amperometric biosensor based on this enzyme was developed by incorporating the mediator Prussian Blue (PB). The biosensor showed a useful detection range in conditions of normal blood/serum glycine concentrations and presented 0.575 nA/µM sensitivity (Wang et al. 2021).

Furthermore, among several characterized homotetrameric FAD-dependent glycine oxidases, as far as we know, there is only one report of a monomeric FAD-dependent GO, identified in *Pseudomonas putida* KT2440 (Equar et al. 2015). This was characterized and presented a higher *K*_m_ and lower *k*_cat_ for glycine than the tetrameric GOs. Additionally, the enzyme showed activity on D-proline, sarcosine, and N-methylglycine. The use of monomeric enzymes opens novel opportunities to improve biosensors by oriented immobilization of the active site (Vazquez-Duhalt et al. 2014), as well as other biotechnological applications such as genetic fusion strategies. *Azotobacter vinelandii* is a soil gram-negative bacterium that has been used to produce polyhydroxybutyrate and alginate (Galindo et al. 2007). The organism belongs to the Gammaproteobacteria class, similar to *P. putida*, the organism that possesses the aforementioned monomeric GO. The amino acid sequence of the GO from *A. vinelandii* presents 75% sequence identity to GOPP and was chosen for this study.

In this work, the GO from *A. vinelandii* CA6 was recombinantly expressed and purified to homogeneity. The oligomeric state and kinetic properties of the enzyme were analyzed, and a biosensor based on the enzyme was assembled and characterized to evaluate its potential application for glycine biosensing.

## Materials and methods

### Genes and cloning

A synthetic gene fragment optimized to express of the amino acid sequence of the glycine oxidase from *A. vinelandii* (AGK18858.1, Genbank) was acquired from the La Proteina Co (CDMX, Mexico). The sequence was amplified by polymerase chain reaction, using the specific oligonucleotides *Avi*GOFw 5’-ATA*CATATG*CGTGTGCTGGTGGTG-3’ and *Avi*GORv 5’-CAT*TCTAGA*TTAACCGATGCGACC-3’, that contain the *Nde*I and *Xba*I restriction sites, respectively (recognition sequences appeared in italic). The amplified product was digested with the restriction enzymes and cloned into the *Nde*I and *Xba*I sites of the pCold I vector.

### Protein expression and purification

The *Avi*GO-pCold I construct was transformed into chemically competent bacteria of the *E. coli* BL21 (DE3) strain. The positive transformants were selected on Luria Broth (LB) agar plates supplemented with 100 µg/mL ampicillin. A single colony of the positive transformants was used to inoculate 150 mL of LB liquid media, containing 100 µg/mL ampicillin. The culture was incubated overnight at 37 °C and agitated at 200 rpm. The overnight culture was used to inoculate 1.5 L of LB medium with ampicillin and incubated at 37 °C and 200 RPM until an OD _600_ ~0.5–0.7 was reached. The culture was cold-shocked in an ice bath for 30 min, then IPTG 200 µM was added, and the incubation continued in a shaker at 20 °C and 200 rpm for 20 h. Bacterial cells were harvested by centrifugation at 6,000 rpm for 10 min. The bacterial pellet was resuspended in 60 mL of binding buffer (Na_2_HPO_4_-NaH_2_PO_4_ 50 mM pH 7.4, 50 mM NaCl, 10 mM imidazole, 2 mM β-mercaptoethanol, and 10% glycerol), 1 mM phenylmethylsulfonyl fluoride, and 1 mg lysozyme were added and incubated for 1 h at 37 °C. The cell suspension was lysed by ultrasonication at 20 kHz, applying cycles of 10 s of sonication and 5 s of resting. Subsequently, the sample was centrifuged at 13,000 rpm for 1 h. The first purification step was performed by injecting the supernatant into a HisTrap column (Cytiva) previously equilibrated with binding buffer. The column was washed with binding buffer, and protein fractions were eluted using a binding buffer containing increasing concentrations of imidazole. The fractions containing the GO were dialyzed against buffer A (50 mM Na_2_HPO_4_-NaH_2_PO_4_ pH 7.4, 50 mM NaCl, 2 mM β-mercaptoethanol, and 10 glycerol) to eliminate imidazole. The dialyzed fraction was injected into a Q-sepharose column previously equilibrated with buffer A. The bound protein was eluted in a linear gradient with buffer B (50 mM Na_2_HPO_4_-NaH_2_PO_4_ pH 7.4, 1 M NaCl, 2 mM β-mercaptoethanol, and 10% glycerol). The protein-containing fractions were evaluated by SDS-PAGE at 12% and protein bands were revealed by Coomassie’s staining.

### *Avi*GO enzymatic characterization

The glycine oxidase activity of the purified enzyme was evaluated by a coupled reaction to horseradish peroxidase (HRP, Sigma Aldrich) according to the protocol described by García-Morales for AvLOx (García-Morales et al. 2023). Briefly, the reaction was performed in 500 µL of 100 mM Tris-HCl pH 8.5, 2 mM 4-aminoantipyrine, 0.04% N, N diethylaniline, and 10 mM glycine. A volume of 5 µL of HRP (0.5 U/µL) was added, and the reaction was initiated by adding the *Avi*GO solution. The concentration of the produced H_2_O_2_ was calculated using the ε_565_ of the quinone diimide compound formed.

For the optimal pH determination, the reaction activity measurement was conducted as described above, but the buffer was substituted with Britton Robinson buffer (40 mM sodium phosphate, 40 mM sodium citrate, and 40 mM boric acid) in a 6.5 to 11 pH range.

For thermal stability, the enzyme was incubated in buffer A (50 mM Na_2_HPO_4_-NaH_2_PO_4_ pH 7.4, 50 mM NaCl, 2 mM β-mercaptoethanol, and 10% glycerol) at 25, 30, 37, and 45 °C. The residual activity was measured after each hour until an accumulated time of 4 h using the reaction mixture previously described. All experiments were performed in triplicate, and the results were plotted using Origin 8.0 (OriginLab).

Saturation kinetics experiments were performed in 500 µL of 100 mM Tris-HCl pH 8.5, 2 mM 4-aminoantipyrine, 0.04% N, N-diethylaniline, and the substrate at different concentrations. A volume of 5 µL of HRP (0.5 U/µL) was added, and the reaction was initiated by adding the purified GO at 20 nM final concentration. The substrate concentrations ranged from 0.5 to 100 mM of glycine or sarcosine. The initial velocities were calculated and plotted *vs.* the substrate concentration. For glycine, the kinetic data was fitted to a substrate inhibition model (Eq. 1), and for sarcosine, a Michaelis-Menten model was used (Eq. 2). Plotting and data analysis was performed using Origin 8.0 (OriginLab). All measurements were performed in triplicate.

Substrate inhibition model used for glycine data:

1 $$\:v=\frac{{V}_{max}\:\left[S\right]}{{K}_{m}+\left[S\right](1+[S]/{K}_{si})}$$

In Eq. 1,$\:v$ refers to the initial transformation rate, $\:{V}_{max}$ is the maximal transformation rate,$\:\left[S\right]$ is the substrate concentration, $\:{K}_{si}$ is the substrate inhibition constant, and $\:{K}_{m}$ is the substrate concentration at half of the maximal velocity.

Michaelis-Menten model used for sarcosine data:

2 $$\:v=\frac{{V}_{max}\:\left[S\right]}{{K}_{m}+\left[S\right]}$$

### Bioelectrode assembly

Carbon paper electrodes were trimmed out to have an electroactive geometric area of 5 × 5 mm. Ten µL of ethanolic Prussian Blue 2.5 mg/mL, as the electrochemical mediator, was drop-casted and left to dry. Then, the purified *Avi*GO was concentrated at 38 mg/mL in PBS 1X buffer, added with 1% glycerol, by using an Amicon ultrafiltration device (Millipore) with a 10 kDa cut-off membrane. This enzyme solution was mixed 1:3 v/v with 1% chitosan in acetic acid 0.5%. Subsequently, 10 µL of the *Avi*GO-chitosan mixture was drop-casted on the previous PB layer, and the bioelectrodes were left to dry. Two different electrodes were prepared from this point: for single-layer bioelectrodes (SLB) a second layer of 10 µL of only chitosan was drop-casted; for two-layer bioelectrodes (TLB) a second layer of the *Avi*GO-chitosan mixture was drop-casted.

## Electrochemical characterization

The electrochemical evaluation was performed using a µStat400 potentiostat (Metrohm Dropsens) with a three-electrode system, a saturated calomel electrode (SCE) as the reference electrode, a platinum wire electrode as the counter electrode, and the working electrode was the assembled bioelectrode. All measurements were performed in 5 mL of 200 mM phosphate buffer (pH 7.4) at 25 °C under continuous agitation using a magnetic stirrer, which properly allowed the diffusion of the analyte.

Chronoamperometry measurements were conducted at a fixed potential of 0 mV, this potential was selected based on preliminary studies that demonstrated the stability of the baseline and the immediate response after the addition of glycine (Figure S5). The steady-state amperometric response of working electrodes at different glycine concentrations was determined by successive glycine additions reaching concentrations from 0.1 to 50 mM. Next, the background current (I1) was determined until a constant current was obtained without the substrate addition (0 mM). Then, after the glycine addition, the steady-state current response was recorded (I2). The obtained current difference (∆I = I2 − I1) was used to plot a calibration curve of ∆I vs. glycine concentration. The steady-state current was obtained by averaging the signal during the 50 s preceding each new glycine injection. Transient current spikes immediately after the injection were excluded from the analysis, as they arise from hydrodynamic disturbances and capacitive charging. All measurements were taken in triplicate.

Nonlinear regression of the Michaelis-Menten model was used to evaluate the apparent electrochemical enzyme kinetics of bioelectrodes toward the substrate, i.e., to describe the steady-state current response (I) as a function of substrate concentration ([S]). This model can be used to study the influence of factors such as temperature, pH, immobilization technique, and diffusion-limiting membranes in an enzyme system (Economou 2013; Mross et al. 2015). The following modified equation was used:

3 $$\:J=\frac{{J}_{max}\:\left[S\right]}{{K}_{mapp}+\left[S\right]}$$

Where J is the current density (µA/cm^2^), *K*_mapp_ (mM) is the apparent Michaelis constant that quantifies the enzymatic affinity for the substrate, and *J*_max_ is the maximum current density (µA/cm^2^). In this case, *J*_max_ represents the maximum apparent current density that the biosensor can provide under conditions where the enzyme layer is saturated with glycine and the electrochemical reduction of H₂O₂ does not limit the rate (Teng et al. 2010; Kalinke et al. 2024).

## Results

### Protein purification and oligomeric state of *Avi*GO

**Fig. 1:**
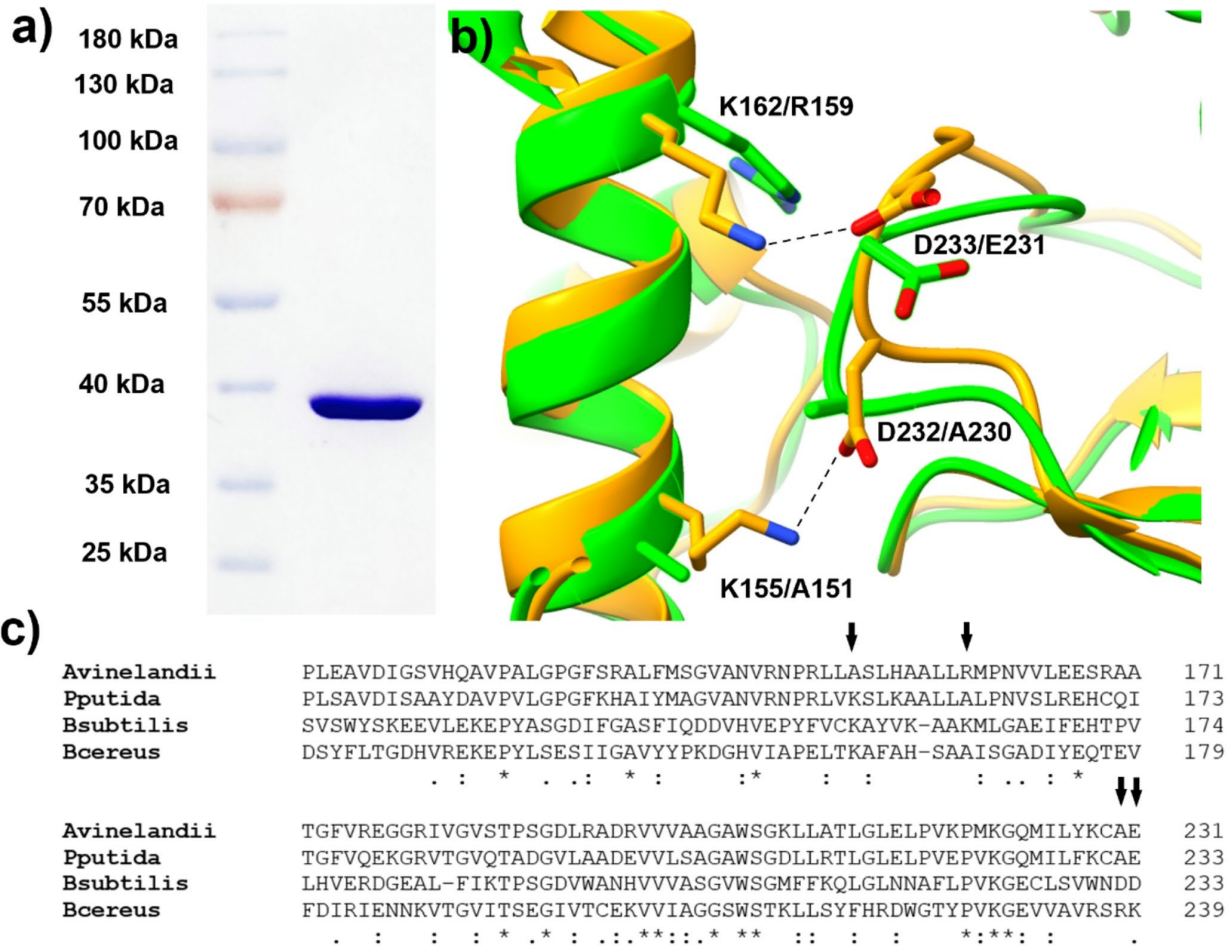
Purified *Avi*GO and tetramer stabilization residues. (**a**) The purified *Avi*GO shows a band with a molecular weight of ~ 40 kDa. (**b**) Superposition of the structure of *Bsu*GO (PDB entry 1NG4, shown as orange ribbons) and the homology model of *Avi*GO shown as green ribbons), the residues that have been proposed to stabilize the tetramer are represented as cylinders and are indicated for *Bsu*GO/*Avi*GO (figure depicted in ChimeraX). (**c**) Sequence alignment of monomeric and tetrameric GOs, the arrows indicate the positions of residues proposed to stabilize the tetramer

*Avi*GO was expressed and purified according to the protocols described in the experimental section. The expression of the protein was evidenced by the presence of an intense band at ~ 40 kDa in the polyacrylamide gels. The *Avi*GO content was enriched in the Ni^2+^ chromatography fractions eluted with imidazole (Figure S1), and a unique band was observed after anionic exchange chromatography (Fig. 1a). The yield of the purified *Avi*GO was 5.8 mg/L of culture. The FAD/*Avi*GO ratio was calculated from UV-visible spectroscopy of the purified enzyme (Figure S2), yielding a ratio of 0.38. This value indicates the fraction of the enzyme that is functional and was subsequently used to properly calculate the molar concentration of the enzyme for further experiments.

To further confirm the enzyme’s molecular weight, another qualitative characterization was performed by size exclusion chromatography, in which the enzyme was eluted at approximately 52 mL. According to the Sephacryl S-100 h 16/60 column protocols, this corresponded to a molecular weight between that of β-lactoglobulin (35 kDa), which eluted at 56 mL, and bovine serum albumin (67 kDa), which eluted at 48 mL. Based on the calculation from its amino acid sequence, the molecular weight of the monomer is 39.4 kDa. In comparison, the molecular weight of the tetramer is 157.6 kDa, suggesting that *Avi*GO is a monomeric enzyme.

Dynamic light scattering (DLS) analysis revealed an average hydrodynamic radius of 3.31 ± 0.59 nm (Figure S3). Compared to the theoretical H_r_ calculated in HullRad (Fleming and Fleming 2018) from the PDB for the tetrameric glycine oxidase from *B. subtilis*, and also for an I-TASSER (Yang and Zhang 2015) homology model for *Avi*GO, the calculated hydrodynamic radius was 4.74 nm for the *B. cereus* tetramer and 2.88 nm for the model of the monomer, respectively. The results of both SEC and DLS analyses confirmed that *Avi*GO is a monomeric glycine oxidase.

The residues responsible for the stabilization of the tetramer were proposed by Equar et al. (2015) after the analysis of *P. putida* GOPP sequence. They proposed two lysine residues from one chain and two aspartate residues from the adjacent chain (Equar et al. 2015), which are paired as K155-D232 and K162-D233 in *Bsu*GO crystal structure (PDB entry 1NG4). The analysis of a sequence alignment of *Avi*GO with GOPP, *Bsu*GO and BceGO (Fig. 1c) and the superposition of the homology model of *Avi*GO with the crystal structure of *Bsu*GO (Fig. 1b) suggest that the absence of the contacts between the proposed pairs of residues (K155 and D232 or K162 and D233) avoids the stable interaction between subunits in *Avi*GO.

### Enzymatic characterization of the *Avi*GO

**Fig. 2:**
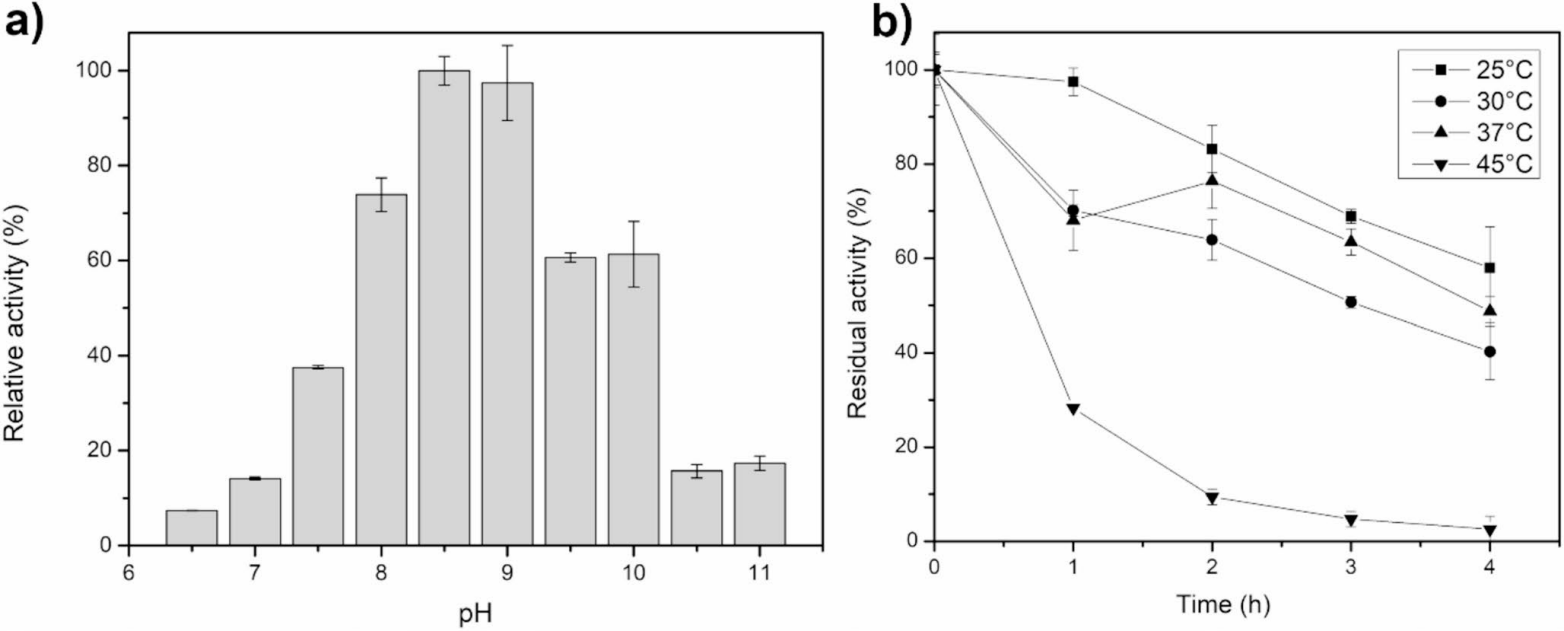
Optimum pH and thermal stability of *Avi*GO. (**a**) pH profile of *Avi*GO. (**b**) Stability of *Avi*GO incubated at different temperatures

The purified *Avi*GO was assayed for activity with glycine as substrate, and its pH activity profile was determined (Fig. 2). The best activity for our enzyme was observed at pH 8.5. However, at a pH of 9, it decreased by only 2.5%. Identically as for *Avi*GO, the reported optimal pH value is 8.5 for all the tetrameric GOs, such as *B. subtilis* (Job et al. 2002), *B.* *licheniformis* (Zhang et al. 2016), *B.* *cereus* (Zhan et al. 2013) *G. kaustophilus* (Martínez-Martínez et al. 2008), and also for GOPP, the only monomeric enzyme found in the literature (Equar et al. 2015). To compare *Avi*GO with these enzymes, and due to the similarity in optimal pH values, all the subsequent enzyme kinetics characterization was carried out at pH 8.5.

*Avi*GO thermostability was assayed after incubation at different temperatures, and residual activity was determined. After 4 h, the enzyme retained 58% of its activity when incubated at 25 °C and 40% at 30 °C, and at 37 °C, the remaining activity was 48%. Finally, for the incubation at 45 °C, the residual activity was 2%.

**Fig. 3:**
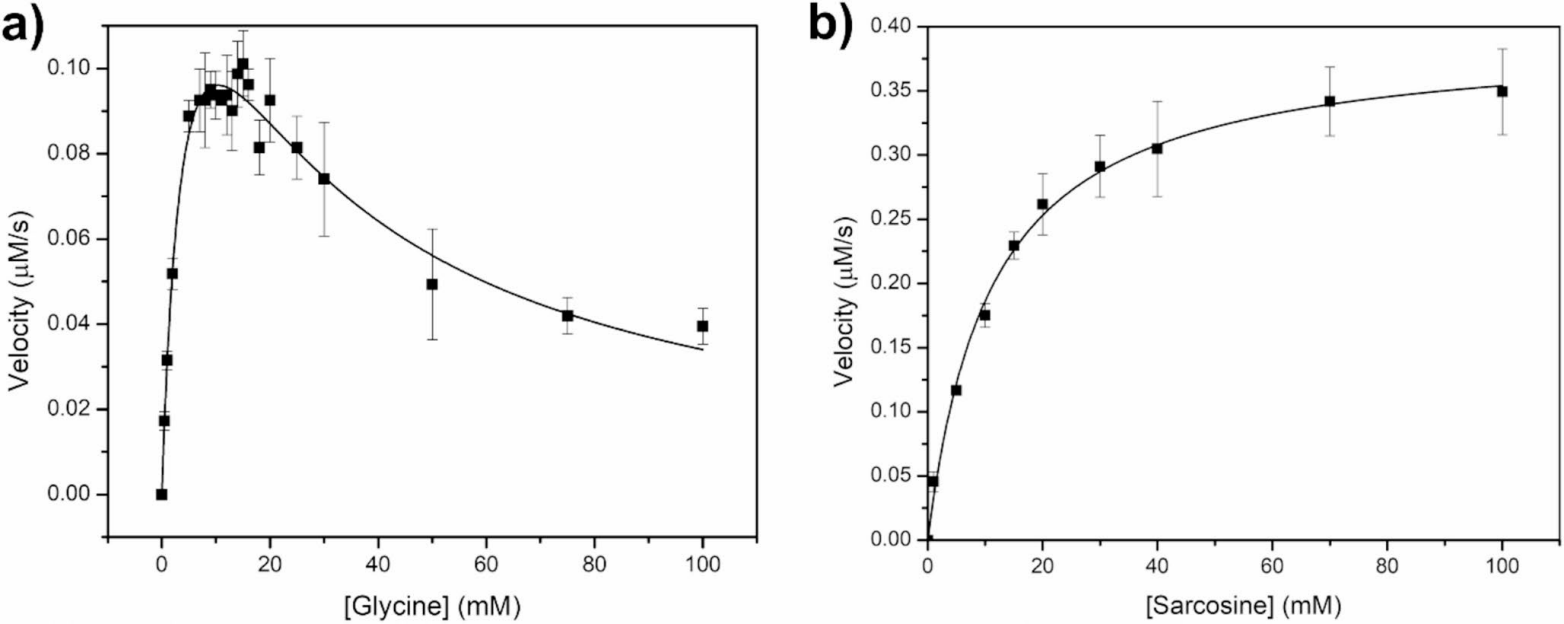
Saturation kinetics of AviGO. Enzyme kinetics using glycine (**a**) and sarcosine (**b**) as substrate. Experimental measurements are presented as solid squares, SD as bars, and the fitting models for the data are presented as a continuous line

Enzyme kinetics experiments were performed with glycine and sarcosine as substrates. For glycine, the υ versus substrate concentration plot was fitted to a substrate inhibition model, while for sarcosine, the inhibition was not observed; thus, the data fitted the Michaelis-Menten model (Fig. 3). The kinetic parameters of *Avi*GO and other reported GOs are presented in Table 1. The *K*_m_ values of *Avi*GO for glycine are higher than those reported for the tetrameric enzymes and also the monomeric PPGO (1.9-fold higher). The *k*_cat_ for glycine (0.38 ± 0.03 s^−1^) is higher than the monomeric enzyme of *P. putida* (0.10 s^−1^), and also that of all the tetrameric FAD-dependent enzymes, except that of *B. subtilis* (0.61 ± 0.03 s^−1^). Regarding the kinetic parameters for the enzyme from *M. mediterranea*, while the *K*_m_ is 0.5 mM, which is very similar to that of the tetrameric FAD-dependent enzymes, the *k*_cat_ is 93 s-1, meaning it is ~ 244-fold faster than *Avi*GO.

**Table 1:** Kinetic parameters of Glycine oxidases

Enzyme	Glycine			Sarcosine			
	K_m_ (mM)	k_cat_ (s^−1^)	k_si_ (mM)	K_m_ (mM)	k_cat_ (s^−1^)	k_si_ (mM)	Reference
*A. vinelandii*	4.76 ± 0.83	0.38 ± 0.03	23.00 ± 3.71	11.09 ± 0.75	0.62 ± 0.01	/	This work
*B. licheniformis*	0.90 ± 0.01	0.31 ± 0.13	/	/	/	/	(Zhang et al. 2016)
*G. kaustophilus HTA426*	0.25	NR	2.78	0.45	NR	5.27	(Martínez-Martínez et al. 2008)
*P. putida KT2440*	2.43	0.10	/	4.86	0.11	/	(Equar et al. 2015)
*B. subtilis*	0.7 ± 0.10	0.61 ± 0.03	/	0.7 ± 0.10	0.60 ± 0.02	/	(Rosini et al. 2014)
*B. cereus* HYC-7	1.04 ± 0.17	0.14 ± 0.1	/	/	/	/	(Zhan et al. 2013)
*M. mediterranea*	0.51 ± 0.42	93 ± 18	/	/	/	/	(Sehanobish et al. 2016)

Regarding the *Avi*GO characterization using sarcosine as substrate, the *K*_m_ and *k*_cat_ values were higher than those of glycine. In comparison with the kinetic parameters for other GOs, the *K*_m_ and *k*_cat_ values have been reported only for *P. putida* and *B. cereus*, in contrast, for *G. kaustophilus V*_max_ is reported instead of *k*_cat_, which makes it difficult to compare *Avi*GO with GOXK.

### Electrochemical evaluation of *Avi*GO-based biosensor

**Scheme 2:**
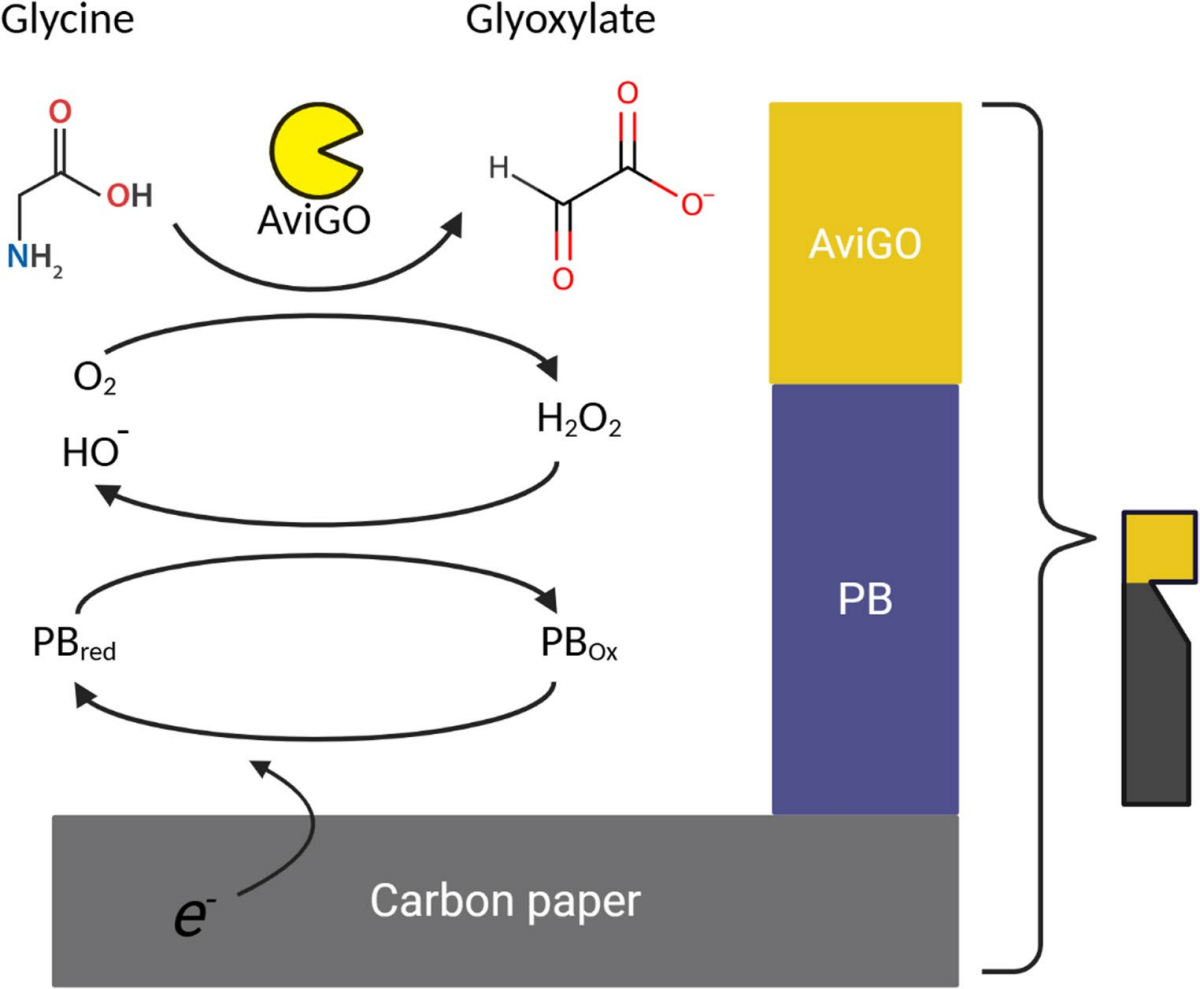
Assembly of the biosensor

The detection mechanism proposed for the two types of bioelectrodes developed in this work (SLB and TLB), which were prepared by varying the number of biorecognition layers, is illustrated in Scheme 2. The initial oxidation of glycine to glyoxylate is catalyzed by the enzyme *Avi*GO to generate H_2_O_2_ This process is divided into two stages: first, glycine donates electrons to FAD within *Avi*GO, reducing it to FADH_2_; then, molecular oxygen accepts electrons from FADH_2_, regenerating FAD and thus forming H_2_O_2_. Meanwhile, at the carbon electrode, the PB deposited is reduced to its Prussian white (PW) form, which is the species that reduces and detects the H_2_O_2_ formed by the *Avi*GO, closing the catalytic cycle by forming the HO^−^ ions (Jiang et al. 2022; Matos-Peralta and Antuch 2019). Thus, glycine is selectively and indirectly detected by the PB-modified carbon-based transducer at a low potential of 0 V against the SCE (Koppenol et al. 2010), generating a cathodic current that varies proportionally to the concentration of the added glycine as can be observed in the cyclic voltammetry of the SLB and TLB biosensors (Figure S4). The developed biosensor can be classified as a second-generation device, since, as extensively documented in the literature, Prussian Blue (PB) functions as a redox mediator (undergoing reversible oxidation–reduction) and effectively acts as an electron carrier between the electrode surface and the hydrogen peroxide generated by *Avi*GO (Chu et al. 2017).

**Fig. 4:**
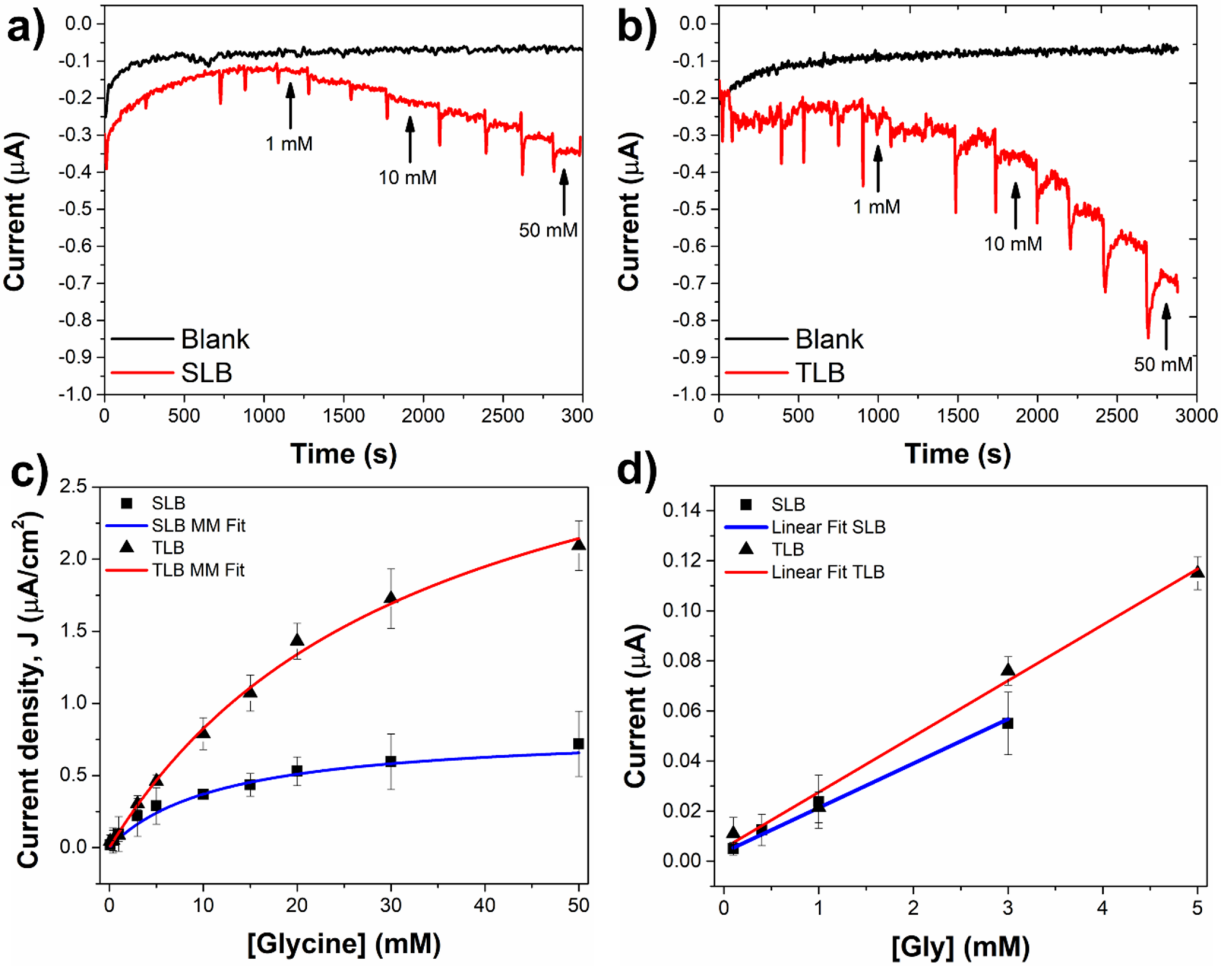
Electrochemical characterization of glycine biosensors based on *Avi*GO. (**a**) Chronoamperometry of SLB, and (**b**) TLB in response to increased glycine concentrations. (**c**) Pseudo-kinetic fit of the ΔI vs. [glycine] for SLB and TLB. (**d**) Determination of the linear range for SLB and TLB

Control experiments revealed that electrodes modified only by PB without *Avi*GO, or *Avi*GO electrodes without PB, did not give a significant analytical signal for the detection of glycine (data not shown). Therefore, both components play an important role in providing the selectivity to the design of this bioelectrode. The two types of bioelectrodes were assayed, and the enzymatic pseudo-kinetic parameters of both SLB and TLB in the glycine detection were obtained. The amperometric measurements were carried out in PBS buffer at pH 7.4, over a concentration range of 0–50 mM at a constant potential of 0 V *vs.* SCE. This potential was selected based on preliminary amperometric studies that demonstrated the stability of the baseline and the immediate response after the addition of glycine (Figure S5). Representative amperometric plots of SLB and TLB are shown in Fig. 4a and b, where there is an evident electrocatalytic signal after successive addition of glycine, obtaining a cumulative ΔI of 0.15 µA and 0.55 µA, respectively, for SLB and TLB at 50 mM glycine, which was the highest concentration assayed for this experiment.

The Michaelis-Menten behavior was observed by plotting the current *vs.* glycine concentration from data of both types of bioelectrodes (Fig. 4c). The pseudo-kinetic parameters J_max_ and *K*_mapp_ were obtained, which turned out to be more favorable for the 2-layer design which obtained a higher current density of 3.57 µA/cm^2^ *vs.* 0.81 µA/cm^2^ of a single layer. TLB also showed a lower *K*_mapp_ of 33.27 mM *vs.* 11.90 mM of SLB. The substrate inhibition observed in solution for *Avi*GO was not observed in the pseudo-kinetic plot of both biosensors.

The ΔI values were plotted versus glycine concentration (Fig. 4d), and the linear range LOD and sensitivity were determined (Table 2). A linear range for TLB was obtained from 0.1 to 5 mM, and from 0.1 to 3 mM for SLB.

**Table 2:** Electrochemical performance of different bioelectrodes based on Glycine oxidases

Bioelectrode	Linear range µM	Sensitivity	LOD	*R* ^2^	reference
**Electrode path- PB-GlyOx @chi-naf** **dimeric quinoprotein** **Marinomonas mediterranea GO**	25–500 µM	Batch 0.881 nA/µM Drop 0.399 nA/µM	11.1 µM 16.4 µM		(Wang et al. 2021 )
**MN-PB-GlyOx** @**Chi-naf** **Microneedle**,** dimeric quinoprotein** **Marinomonas mediterranea GO**	25–600 µM	−0.147 nA/µM	7.9 µM	-	(Wang et al. 2022 )
**GlyOx-266TF-Bodipy373/CNT/GCE** **Tetrameric** **Bacilus subtilis** **wiring**		Sweat sample Reference value (mM) from LC-MS 1.22 ± 0.05 Measured value (mM) from Amperometry 1.34 ± 0.10			(Xia et al. 2019 )
**SLB** **Monomeric** ***Avi*** **GO**	100–3000	16.8 nA/mM	0.126 mM	0.9974	This study
**TLB** **Monomeric** ***Avi*** **GO**	100–5000	16.2 nA/mM	0.421 mM	0.9983	This study

To evaluate the potential applicability of the *Avi*GO-based biosensor in biological matrices, typical serum interferents (ascorbate, glucose, lactate, urea, and human serum albumin) were assayed at physiological concentrations (see Supplementary Information, Figure S6). In the presence of this mixture, the single-layer biosensor (SLB) retained its amperometric response to glycine, exhibiting a linear calibration with a sensitivity of 31.16 nA mM⁻¹ over 1–5 mM (R² = 0.9928), compared to 16.8 nA mM⁻¹ over 0.1–3 mM in buffer (R² = 0.9974). Although the linear range shifted to higher concentrations under the interferent matrix, the sensitivity remained of the same order of magnitude, indicating no significant loss of analytical performance.

## Discussion

### *Avi*GO is a monomeric enzyme

The crystal structure of several tetrameric GOs has been reported for *B. subtilis* (PDB entry 1NG4) (Settembre et al. 2003), *B.* *cereus* ATCC 14,579 (PDB entry 7CYX) (Seok et al. 2020) *G.* *kaustophilus* (PDB entry 4YSH). In all of them, the residues proposed for stabilization of the tetramer are conserved. However, those interactions are absent in PPGO, which possesses K153-A232 and L150-E233 (Equar et al. 2015), instead of K155-D232 and K162-D233 of *Bsu*GO. In *Avi*GO both pairs are substituted for A151-A230 and R159-E231, even if these last pair of residues would present charge complementarity. The homology model suggests that the orientation of side chains is not compatible with electrostatic interactions, which make difficult to stabilize the dimer and also the tetramer.

Regarding applications in biosensors, the monomeric enzymes could be useful for oriented immobilization in electrode surfaces, avoiding common problems of multimeric enzymes, where the active sites commonly point in different directions (Arrocha et al. 2014; Vazquez-Duhalt et al. 2014). For example, in tetrameric glycine oxidases, the active sites of individual monomers are rotated relatively to each other, making it impossible to orient all sites toward an electrode surface at the same time. The confirmation of the monomeric state of the *Avi*GO is pivotal for the further development of the glycine biosensor.

### Enzymatic properties of *Avi*GO

The optimal pH for *Avi*GO activity was determined to be 8.5, with a progressive decrease in catalytic activity observed at lower pH values. This decrease in enzymatic activity at pH values below 8.5 could be a disadvantage for the *Avi*GO application in glycine biosensing. Different pH values can be found in physiological fluids. Still, all of them are below the optimal pH of *Avi*GO, i.e., in blood serum, the pH is ~ 7.4 (where *Avi*GO activity is 37%), and in sweat, where pH oscillates between 4.1 and 5.9. The activity was not measured at these pH values; however, at pH 6.5 it was 7%, suggesting that it would tend toward zero at lower pH values.

Given the low stability of the enzyme at room temperature, its applicability could be limited, as biosensors are often operated at ambient temperature or at 37 °C. Nevertheless, several strategies are available to enhance enzyme performance in terms of pH tolerance and thermostability, including the use of chemical additives, protein engineering, and immobilization techniques (Beaufils et al. 2021).

After kinetic characterization of *Avi*GO and comparison of parameters with other characterized GOs, it is important to highlight that the kinetic mechanism for GOs was proposed for *B. subtilis GO*, and involves the hydride transfer from Cα of glycine to N5 of the isoalloxazine moiety of FAD. The residues R298, R327, and N328 are conserved in the other characterized GOs and are involved in the proper binding of glycine (Settembre et al. 2003; Wu et al. 2016). Several reports describe mutants of *B. subtilis* or *B. licheniformis* glycine oxidases; in some of them, the *K*_m_ is increased and *k*_cat_ is similar to *Avi*GO. For example, in the H244A mutant of *B. subtilis* GO, the *K*_m_ increased from 0.34 to 1.5 mM, compared to wild-type, and in M261L, the resulting *K*_m_ was 1.3 mM (Boselli et al. 2007). In *Avi*GO, the residues equivalent to H244 and M261 are glycine and leucine, respectively, according to the homology model, and it could be possible that these differences in the active sites of the enzymes are at least partially responsible for the increased *K*_m_ in the monomeric GOs described so far. As can be inferred from the model and the sequence comparison between GOs, several other differences in the residues that form the active site of the enzymes would require structural studies to perform appropriate comparisons. Regarding the enzyme from *P. putida*, which is also monomeric, lower *k*_cat_ and *K*_m_ values than *Avi*GO are reported. Additionally, only one other enzyme has been reported to exhibit substrate inhibition for glycine: the GO from *G. kaustophilus* (Martínez-Martínez et al. 2008), which also displays substrate inhibition with sarcosine. In contrast, the saturation kinetics *Avi*GO, with sarcosine, can be accurately fitted to the Michaelis-Menten model. Currently, there is insufficient information to elucidate the structural features underlying the substrate inhibition mechanism or to identify the residues involved. However, the differences in enzyme kinetic models for both substrates suggest a specific strong regulation in activity for glycine. These findings point toward glycine as the physiological substrate of *Avi*GO. Further structural characterization by X-ray crystallography or molecular dynamics with a homology model would help understand the specificity for the substrate inhibition of *Avi*GO using glycine as substrate.

### *Avi*GO-based biosensor and potential application

Substrate inhibition was not observed at high glycine concentrations in the biosensors (Fig. 4). The immobilized enzymes in the chitosan-enzyme composite could experience constraints in mobility and modified diffusion of substrates through the bioelectrode (Mateo et al., 2007; Bai et al. 2023). The lack of substrate inhibition in the biosensor suggests a limited access of the substrate to the site responsible for enzyme inhibition, or a limitation of conformational changes that could be necessary for the inhibition. In any case, neither the kinetic nor the electrochemical results are enough to explain the mechanism for substrate inhibition specifically.

Few works are available in the literature on electrochemical biosensors based on GO for glycine detection (Table 2). Wang et al. (2022) reported a biosensor based on the dimeric *Marinomonas mediterranea* GO dependent on the tryptophyl quinone cofactor, first by developing a path with the direct electrodeposition of PB, followed by the modification of chitosan-entrapped GO covered with a Nafion layer, and applied to a microneedle (Wang et al. 2022). The developed biosensors presented a linear range between 25 and 500 µM glycine, LOD of 11.7µM, and a sensitivity of 0.575 nA/µM, which are consistent with normal glycine levels in physiological fluids such as blood, plasma, or cerebrospinal fluid. However, this approach represents an invasive procedure that should be avoided in the new technologies.

On the other hand, Xia et al. (2019) obtained mutants of the tetrameric GO of *B. subtilis*, and the bioelectrode based on direct wiring to a CNT-modified GCE electrode showed determinations comparable to those obtained by LC-MS up to 0.12 mM in sweat samples (Xia et al. 2019). They highlight the need for new procedures, including the use of nanomaterials and direct wiring for signal enhancements. Moreover, applying targeted mutation to obtain favorable results in the determination of glycine by GO-based biosensing.

As described above, the kinetic parameters of the non-FAD enzymes are more appropriate for detecting low concentrations of glycine in physiological fluids than those from the FAD-dependent enzymes. It seems that FAD-dependent monomeric enzymes can be applied to non-invasive situations, such as the glycine determination in urine or sweat samples, where concentrations are higher.

The glycine biosensors assembled in this work showed a sensitivity of 16.8 and 16.2 nA/mM with a linear range of 0.1–3.1 mM and 0.1 to 5 mM for SLB and TLB, respectively. The linear ranges are much higher than those previously reported. The SLB could be useful to evaluate glycine in physiological concentrations in sweat (1759 ± 150 µM) or abnormal concentrations in urine (550–5000 µM) (Pérez-Ràfols et al. 2020). Meanwhile, the TLB is useful for the range of abnormal glycine concentration in urine. However, improving the linear range of both biosensors for the physiological fluids is necessary, and it is difficult to achieve using the wild-type. In previous reports, GO mutants from *B. subtilis*, *B. cereus*, or *B. licheniformis* with lower *K*_m_ and higher *k*_cat_ have been obtained. Thus, mutation of *Avi*GO could improve the linear range for the detection of glycine. As previously mentioned, elucidating the structure of *Avi*GO would provide valuable insights for the rational design of mutants with enhanced biosensing performance. Nevertheless, the straightforward design implemented here, based on drop-casting deposition techniques, represents a practical and easily scalable approach for developing non-invasive technologies that demand simple fabrication processes, such as wearable biosensors. On the other hand, some strategies to improve the electrode’s sensitivity should be considered, such as the pH modification of the buffer for measurements. The mediator PB is a reliable reductor for H_2_O_2_, and there are reports where the *in situ* synthesis of PB gives high stability and signal on alkaline pH 8.0 (Moscone et al. 2001; Ricci et al. 2003). At this pH, *Avi*GO maintains 70% of its activity when compared to optimal pH, whereas at pH 7.5, the activity is only 35%. The hypothetical 2-fold increase in signal that could be obtained if PB is stabilized at alkaline pH could improve the performance of the *Avi*GO-based biosensor.

Regarding the biosensor response against interferent molecules, this is particularly relevant because Prussian blue–based electrodes are often reported to display cross-reactivity toward electroactive species such as ascorbate and urate, potentially compromising selectivity (Zloczewska et al. 2014). For the *Avi*GO biosensors, embedding the enzyme within the chitosan/PB matrix preserved signal integrity in the presence of interferents, suggesting sufficient robustness for operation in complex biological environments. Together, these observations support the applicability of the proposed biosensor for detecting elevated glycine levels in clinically relevant fluids such as blood serum, where interfering species are unavoidable.

## Conclusion

The GO from * A. vinelandii* is a monomeric enzyme; although it shows a lower affinity for glycine than the tetrameric enzymes, it exhibits a higher *k*_cat_ when compared to the FAD-dependent GOs, except for * B. subtilis*. The obtained glycine biosensor exhibits a linear range that is not suitable for detecting normal glycine levels in clinical blood or cerebrospinal fluid samples; however, it can be useful for quantification of abnormal glycine concentrations in blood, urine, and other physiological samples, such as sweat.

To the best of our knowledge, this study represents the first investigation into the potential application of a monomeric GO enzyme for glycine detection, targeting the integration into advanced wearable biosensors that employ non-invasive sampling techniques. Additional strategies for the rational design of mutant enzymes and the *in situ* synthesis of PB could further improve the biosensor’s performance for detecting glycine at low concentrations.

## Supplementary Information

Below is the link to the electronic supplementary material.

Supplementary Material 1

## Data Availability

Data is provided within the manuscript or supplementary information files.
