# Supplementary material for: Monomeric Glycine oxidase from Azotobacter vinelandii for Glycine biosensing
Source: World J Microbiol Biotechnol. 2025 Nov 11;41(11):449. doi: 10.1007/s11274-025-04657-4 (PMC12605379; doi:10.1007/s11274-025-04657-4)
Supplement: Supplementary file 1 — Supplementary Material 1 [file 11274_2025_4657_MOESM1_ESM.docx]

**A monomeric Glycine Oxidase from *Azotobacter vinelandii* with the Potential for Glycine Biosensing**

**Aaron Mena-Rodríguez^a^, Raul García-Morales^a^, Oscar González-Davis^a^, Rafael Vázquez-Duhalt^a^, Alejandro Huerta-Saquero^a^, Andrés Zárate-Romero^a,b,*^**

^a^Centro de Nanociencias y Nanotecnología, Universidad Nacional Autónoma de México, Ensenada, B. C. 22800, México.

^b^Secretaría de Ciencia, Humanidades, Tecnología e Innovación, Alc. Benito Juárez, CDMX 03940, México

*Correspondig author at Tijuana-Ensenada road km 107, Ensenada, B.C. 22800, México

e-mail: azarate@ens.cnyn.unam.mx

**SUPPLEMENTARY FIGURES**

**
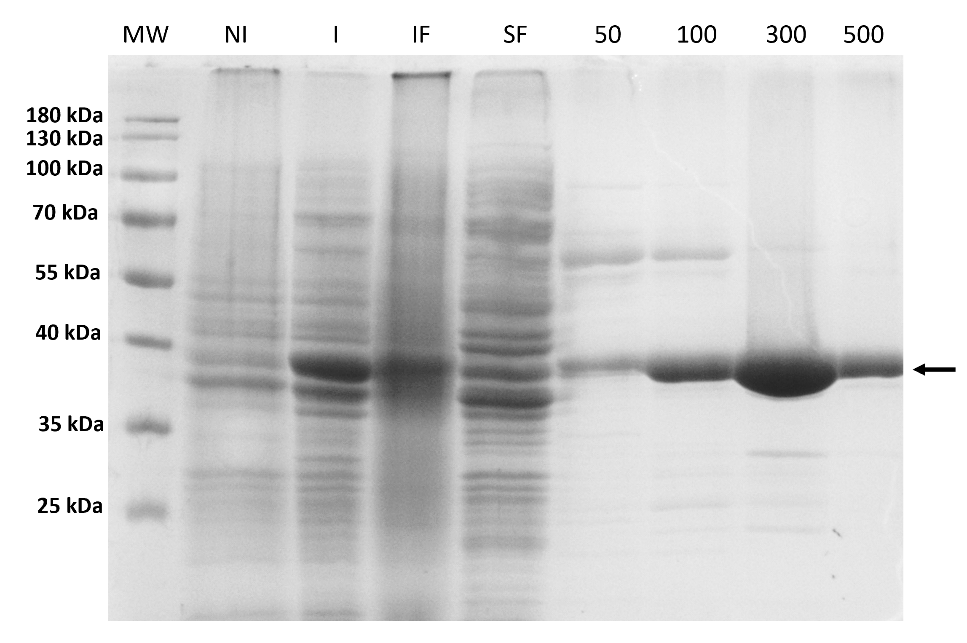
**

**Figure S1.** SDS-PAGE of AviGO expression and Ni^2+^ affinity purification. The position of the expected band for AviGO is shown with the arrow. Each lane corresponds to: MW, protein ladder; NI, non-induced; I, induced; IF, insoluble fraction; SF, soluble fraction; 50, 100, 300, and 500 are the imidazole concentrations (mM) of the buffers used to elute the fractions.

**
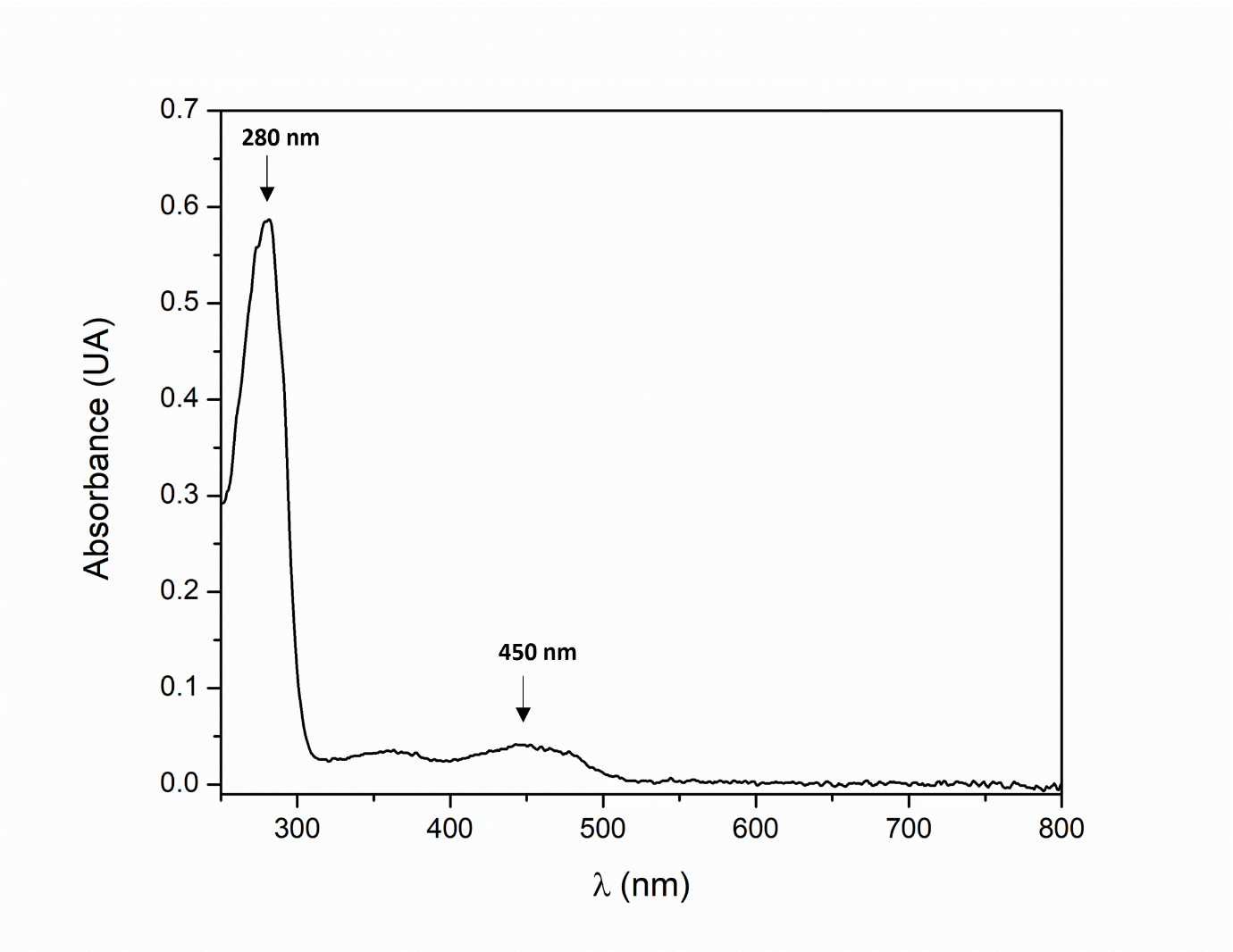
**

**Figure S2.** UV-visible spectrum of the purified AviGO. The arrows indicate the absorption bands at 280 nm and 450 nm.


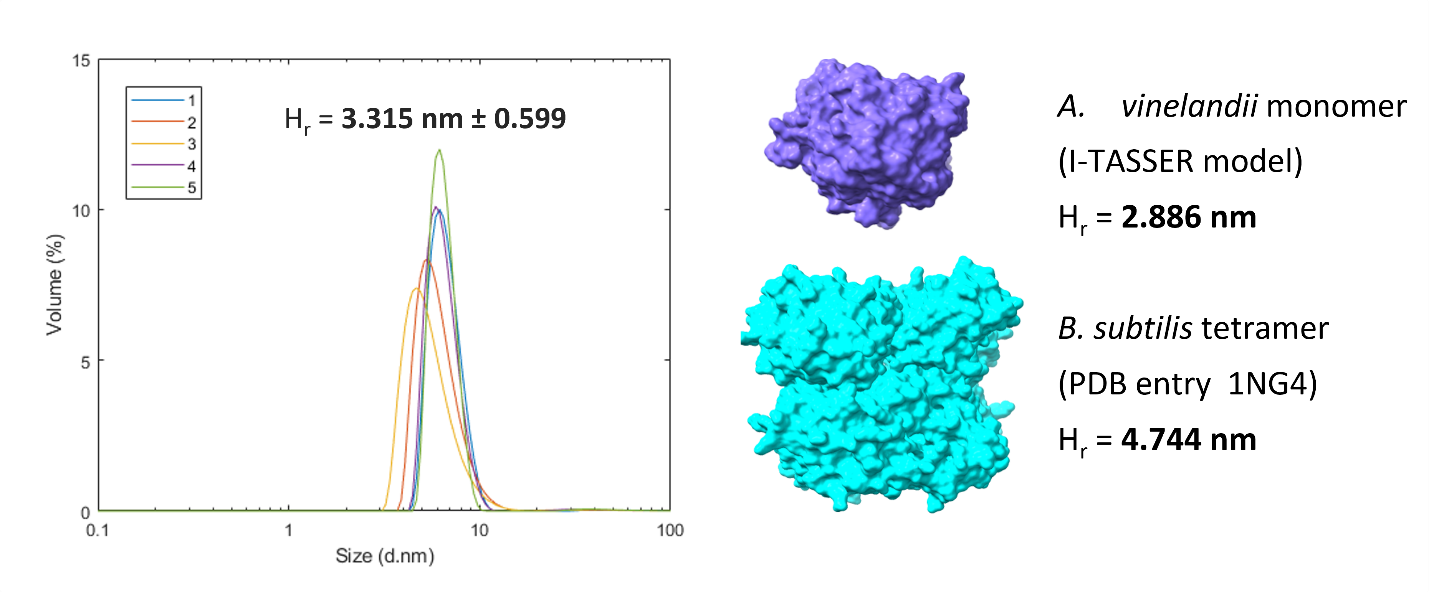


**Figure S3.** Oligomeric state determination of AviGO. a) DLS determination of H_r_. b) Theoretical H_r_ from three-dimensional structures.


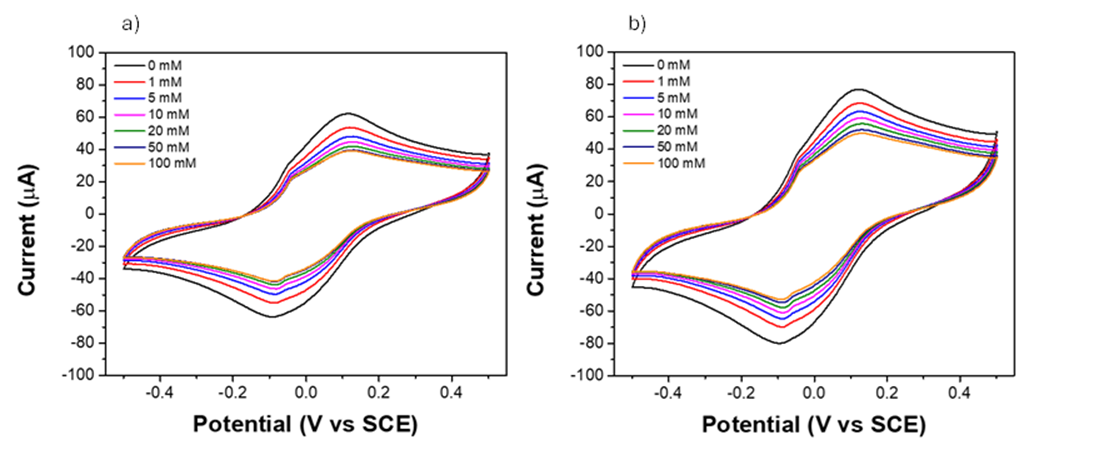


**Figure S4**. Cyclic voltammograms of the AviGO biosensors. a) SLB cyclic voltammogram. b) TLB cyclic voltammogram. The SLB and TLB cyclic voltammograms revealed the presence of a cathodic peak at -100 mV and an anodic peak at 100 mV in the absence and the presence of different glycine concentrations; these peaks are associated with those reported for the oxidation and reduction of the mediator Prussian Blue. The cyclic voltammograms of the AviGO–PB bioelectrode show a progressive decrease in current with increasing glycine concentration. This behavior is attributed to oxygen depletion at the enzyme–electrode interface, which limits PB regeneration, together with mass-transport restrictions imposed by the porous carbon paper and chitosan matrix. The resulting confinement of substrates and accumulation of enzymatic byproducts further disturb the redox balance of PB, leading to current attenuation, as similarly reported for PB-based biosensors.


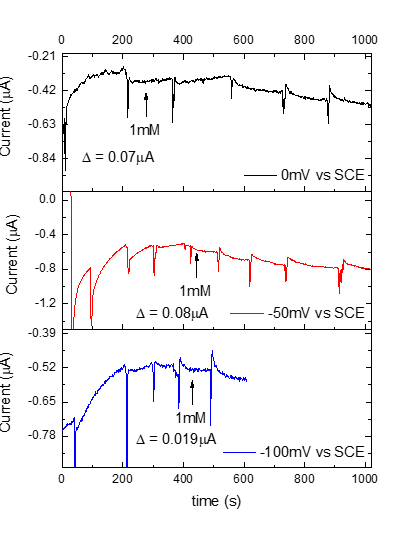


**Figure S5.** Preliminary studies to determine glycine biosensing potential vs SCE. The study was performed using SLB type electrodes in PBS.


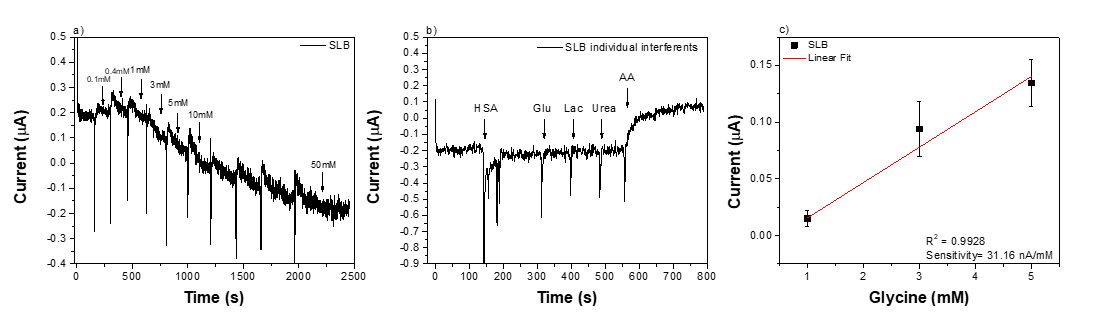


**Figure S6.** Amperometric responses of the AviGO-based biosensor in the presence of common interferents. (a) Amperogram recorded for successive glycine additions in a solution containing physiological concentrations of human serum albumin (35 g/L), glucose (5 mM), lactate (250 µM), urea (200 µM), and ascorbic acid (100 µM). (b) Individual amperometric responses obtained for each interferent in the absence of glycine, showing their minimal contribution to the total signal. (c) Calibration plot derived from (a). Measurements were triplicated and carried out at 0V vs SCE.

**SUPPLEMENTARY METHODS**

**UV-visible spectroscopy of the purified protein**

The purified protein was concentrated and stored in buffer A containing 50 mM Na_2_HPO_4_-NaH_2_PO_4_ pH 7.4, 150 mM NaCl, 10% glycerol, and 2 mM β-mercaptoethanol. The UV-visible spectra of *Avi*GO were acquired in a NanoDrop 2000C spectrophotometer (ThermoScientific). The calculation of the concentrations was performed using the extinction coefficient of 11,300 M^-1^cm^-1^ for FAD (Pedotti et al., 2009) and 55,920 M^-1^cm^-1^ for the *Avi*GO monomer.

**Evaluation of the AviGO oligomeric state**

Size exclusion chromatography (SEC) of the purified protein was performed on a Sephacryl S-100 HR 16/60 column (Cytiva) at 0.5 mL/min flow rate in a buffer solution containing 50 mM Na_2_HPO_4_-NaH_2_PO_4_ pH 7.4, 150 mM NaCl, 10% glycerol, and 2 mM β-mercaptoethanol, and the retention time was analyzed. After the elution, the purified protein was filtered, diluted, and sonicated in a bath ultrasonicator. The hydrodynamic radius of the protein in the fraction was determined by dynamic light scattering (DLS) in a Zetasizer nano (Malvern panalytical).

**Homology model and prediction of hydrodynamic radius**

A homology model was obtained for the AviGO monomer using the server I-Tasser (Yang & Zhang, 2015). The resulting model was minimized and was used to calculate the hydrodynamic radius (H_r_) in the HullRad (Fleming & Fleming, 2018) server. For the H_r_ calculation of the tetramer, the coordinates of the *B. subtilis* GO (PDB entry 1NG4) were analyzed in the HullRad server.

**Electrochemical characterization**

The electrochemical experiments were performed in a μStat400 potentiostat (Metrohm Dropsens) with a three-electrode system, a saturated calomel electrode (SCE) as the reference electrode, a platinum wire electrode as the counter electrode, and the working electrode was the assembled bioelectrode. All measurements were performed in 5 mL of 200 mM phosphate buffer (pH 7.4) at 25°C under continuous agitation using a magnetic stirrer. The cyclic voltammetry experiments were conducted at a potential from 0.5 to -0.5 V at a sweep rate of 100 mV/s at different glycine concentrations in the range 0-100 mM. The preliminary amperometric measurements were performed at 0, -50, and -100 mV. The glycine concentrations were increased to evaluate the current at the different potentials.

For the interferent assay, a buffer solution containing: 35g/L human serum albumin, 5 mM glucose, 250 μM L-lactate, 200 μM urea, and 100 μM ascorbic acid was prepared. After the addition of increasing glycine concentrations, the current intensity was measured. A second experiment was performed by sequentially adding the interferents to the buffer to determine the contribution of each single interferent to the ΔI.

**REFERENCES**

Fleming, P. J., & Fleming, K. G. (2018). HullRad: Fast Calculations of Folded and Disordered Protein and Nucleic Acid Hydrodynamic Properties. *Biophysical Journal*, *114*(4), 856–869. https://doi.org/10.1016/j.bpj.2018.01.002

Pedotti, M., Rosini, E., Molla, G., Moschetti, T., Savino, C., Vallone, B., & Pollegioni, L. (2009). Glyphosate Resistance by Engineering the Flavoenzyme Glycine Oxidase. *Journal of Biological Chemistry*, *284*(52), 36415–36423. https://doi.org/10.1074/jbc.M109.051631

Yang, J., & Zhang, Y. (2015). I-TASSER server: New development for protein structure and function predictions. *Nucleic Acids Research*, *43*(W1), W174–W181. https://doi.org/10.1093/nar/gkv342
